# Supplementary material for: Interplay of Modifiable and Non-Modifiable Risk Factors for Diabetes Mellitus in Saudi Adults
Source: Diagnostics (Basel). 2025 Sep 25;15(19):2451. doi: 10.3390/diagnostics15192451 (PMC12523265; doi:10.3390/diagnostics15192451)
Supplement: Supplementary file 1 [file diagnostics-15-02451-s001.zip › diagnostics-3872702-supplementary.pdf]

Diabetes Mellitus Questionnaire

Table S1. This questionnaire is designed to assess lifestyle, dietary, and health-related factors among adults in Saudi Arabia (DM part). Please answer all questions. Your responses will remain confidential.

**Section**	**Question**	**Response Options**
Sociodemographic Information	1. Age	____ years
	2. Gender	☐ Male ☐ Female
	3. Education level	☐ Illiterate ☐ Elementary ☐ Intermediate ☐ Secondary ☐ University ☐ Postgraduate
	4. Marital status	☐ Single ☐ Married ☐ Divorced ☐ Widowed
	5. Monthly income (SAR)	☐ <5,000 ☐ 5,000–10,000 ☐ 10,000–15,000 ☐ >15,000
	6. Residence	☐ Urban ☐ Rural
	7. Housing	☐ Rented ☐ Owned apartment ☐ Owned traditional house ☐ Owned villa
	8. Family size	____ members
Lifestyle Habits	9. Smoking	☐ No ☐ Yes, cigarettes ☐ Yes, shisha
	10. Physical activity	☐ None ☐ <30 min, 5x/week ☐ ≥30 min, 5x/week
	11. Sleep hours	____ hours per night
Dietary Habits	12. Whole grain products	☐ Yes ☐ No
	13. Fruits & vegetables (≥5 servings/day)	☐ Yes ☐ No
	14. Low-fat meats (e.g., skinless chicken, lean beef/lamb)	☐ Yes ☐ No
	15. Avoidance of sugary foods	☐ Yes ☐ No
	16. Low-fat products (e.g., milk, yogurt, cheese)	☐ Yes ☐ No
Medical History	17. Physician-diagnosed diabetes mellitus	☐ Yes ☐ No
	18. Family history of diabetes mellitus	☐ Yes ☐ No
	19. Family history of dyslipidemia	☐ Yes ☐ No
	20. Height	____ cm
	21. Weight	____ kg
